# The cholinesterase inhibitor donepezil has antidepressant-like properties in the mouse forced swim test

**DOI:** 10.1038/s41398-020-00928-w

**Published:** 2020-07-25

**Authors:** Paul J. Fitzgerald, Pho J. Hale, Anjesh Ghimire, Brendon O. Watson

**Affiliations:** Department of Psychiatry, University of Michigan, Ann Arbor, MI 48109 USA

**Keywords:** Psychiatric disorders, Neuroscience

## Abstract

Finding new antidepressant agents is of high clinical priority given that many cases of major depressive disorder (MDD) do not respond to conventional monoaminergic antidepressants such as the selective serotonin reuptake inhibitors (SSRIs), tricyclic antidepressants, and monoamine oxidase inhibitors. Recent findings of effective fast-acting antidepressants indicate that there are biological substrates to be taken advantage of for fast relief of depression and that we may find further treatments in this category. In this vein, the cholinergic system may be a relatively overlooked target for antidepressant medications, given its major role in motivation and attention. Furthermore, the classically engaged monoaminergic neurotransmitter systems in depression treatment—serotonin, norepinephrine, and dopamine—interact directly at times with cholinergic signaling. Here we investigate in greater detail how the cholinergic system may impact depression-related behavior, by administering widely ranging doses of the cholinesterase inhibitor drug, donepezil, to C57BL/6J mice in the forced swim test. First, we confirm prior findings that this drug, which is thought to boost synaptic acetylcholine, promotes depression-like behavior at a high dose (2.0 mg/kg, i.p.). But we also find paradoxically that it has an antidepressant-like effect at lower doses (0.02 and 0.2 mg/kg). Further this antidepressant-like effect is not due to generalized hyperactivity, since we did not observe increased locomotor activity in the open field test. These data support a novel antidepressant-like role for donepezil at lower doses as part of an overall u-shaped dose-response curve. This raises the possibility that donepezil could have antidepressant properties in humans suffering from MDD.

## Introduction

Major depressive disorder (MDD) is a debilitating neuropsychiatric disorder that is a significant public health problem throughout the world^1,2^. While depression has been the focus of intensive research efforts by a large number of scientists for decades, many cases of this disorder remain resistant to existing behavioral and pharmaceutical therapeutics^3^. Developing new effective treatments, including pharmacological ones, is therefore of high interest and urgency for the field of psychiatry. Most of the drugs used by clinicians to treat depression (selective serotonin reuptake inhibitors (SSRIs), serotonin–norepinephrine reuptake inhibitors, norepinephrine–dopamine reuptake inhibitors, tricyclic antidepressants, and monoamine oxidase inhibitors) are thought to act primarily via the monoaminergic neurotransmitters: serotonin, norepinephrine, and dopamine. While these classes of drugs are effective antidepressants for many cases of MDD, other cases show only a partial response or no response to them, possibly due to different underlying mechanisms of the depressive state in these cases. Furthermore, most antidepressant medications require weeks to begin taking effect^4^, creating a clinical demand for new types or classes of antidepressants, especially those with rapid effects.

Since most monoaminergic antidepressants are thought to at least partially act through raising the synaptic levels of serotonin, norepinephrine, and/or dopamine, one possibility is that novel, small molecule antidepressants might be synthesized (or already exist) which interact with these three neurotransmitter systems in some unique way. One alternative brain signaling molecule, the neurotransmitter acetylcholine, interacts with the three monoamine systems in a number of ways in the brain^5,6^. And while the conventional thinking about the three monoaminergic systems is that they may each have distinct functional properties (e.g., serotonin: appetite/digestion and cognitive flexibility; norepinephrine: stress response and attention/alertness; dopamine: reward, learning, and movement), they may to some degree be functionally overlapping, including in their effects on mood^6^. By extension, acetylcholine, which has a spatially overlapping brain distribution with the monoamines (including prefrontal cortex) and has receptors on many of the same neurons that they innervate^7^, may not only interact with these systems but also share functional properties with them, including mood regulation. On the other hand, another possibility is that acetylcholine makes a unique contribution to mood regulation, given that its behavioral, physiological, and neuroanatomical characteristics, such as strong association with attention, cognition^8^, learning, and dense innervation of layer 1 neocortex, differ in some ways from the monoamines.

There are conflicting data, both in humans and rodents, on whether pharmacological boosting of acetylcholine is pro-depressant or antidepressant. Here we briefly outline some of these findings, beginning with pro-depressant results. Most research into this question has used relatively high dose pro-cholinergic drugs, both in humans and in animal models, and has tended to support this hypothesis^9^. For example, the cholinesterase inhibitor, physostigmine, increases depression-like behavior in the forced swim test (FST) or tail suspension test in mice^10–15^. Consistent with these findings in rodents, clinical administration of cholinesterase inhibitors to individuals with mood disorders has shown depression-promoting effects, and in some cases attenuation of mania or hypomania (e.g., refs. ^14–16^). On the other hand, a number of studies have shown antidepressant-like effects of elevating acetylcholine. A 2016 rat chronic stress study found that chronically administered cholinesterase inhibitors are antidepressant-like in the sucrose preference test^17^. An FST study of Swiss mice found that donepezil, a widely clinically used cholinesterase inhibitor, dose-dependently reduced immobility (i.e., an antidepressant-like effect)^18^. A study of olfactory bulbectomized mice found that the cholinesterase inhibitor rivastigmine increases sucrose preference and reduces immobility in the FST^19^. A number of studies have also shown that the cholinergic receptor agonist, nicotine, has antidepressant-like properties in rodents^20–23^. Since cholinesterase inhibitors increase synaptic cholinergic signaling, presumably at both muscarinic and nicotinic receptors, these drugs may have antidepressant-like properties by boosting activity at either class of receptors. In addition, several case report studies in human subjects have shown that cholinesterase inhibitors can induce mania or hypomania rather than depression^24–27^.

Given these discrepant data in the literature, possibly as a function of dose, and the potential translational impact of repurposing a cholinesterase inhibitor such as donepezil for use in human mood disorders, we were motivated to systematically investigate the behavioral effects of a wide range of doses of donepezil in the mouse FST. This single integrated study using a standard assay of depression-related behavior in rodents may better illuminate these apparently discrepant findings as part of a larger picture^28^. Furthermore, given the chronic nature of some of the studies showing antidepressant responses, we did repeated dosing with repeated testing for each dose. Finally, to determine more broadly how varying doses of donepezil affect behavioral state, we also tested for general locomotor and anxiety-related effects of donepezil in the open field test (OFT).

## Methods

### Subjects

One hundred sixty (*n* = 8 per drug cohort) experimentally naive adult (8–9 weeks old upon arrival) male C57BL/6J mice were obtained from a commercial supplier (The Jackson Laboratory, Bar Harbor, ME). We used this moderate sample size (*n* = 8) because mouse FST experiments of antidepressant compounds, such as imipramine, fluoxetine, or bupropion, typically have reported relatively large effect sizes (>1.0) and often used this approximate sample size per group^29–31^. We used a random number generator to assign animals to drug groups. No blinding of the experimenter to the drug groups was carried out because we used an automated behavioral scoring procedure (see below). Upon arrival and throughout the experiments, mice were group housed in cages within a humidity- and temperature-controlled vivarium, and kept on a 12:12 h light/dark cycle (lights on at 6 a.m.) with ad libitum access to food and water. Each cage had an Enviropak (Lab Supply, Fort Worth, TX) for enrichment and use in nest building. All mice were handled daily by the experimenter (for ~30 s per day) for the first 5 days upon arrival, to acclimate them to the experimenter (PJF). The experimenter also tailmarked the mice upon arrival, and re-tailmarked them every 3 days throughout the experiments. All experiments were carried out in the daytime during the light phase. The first behavioral test of each experiment (Day 0) was carried out 1 week after the mice arrived in our facility. All procedures were conducted at the University of Michigan and were performed in strict accordance with the guidelines and regulations set forth by the National Institutes of Health and the University of Michigan, with full approval from its Institutional Animal Care and Use Committee (Protocol number: PRO00007803). The five experiments described here, while related to one another and having some degree of overlapping information, were not repeated.

### Drug

Donepezil (Cayman Chemical, Ann Arbor, MI) was dissolved in a vehicle (VEH) solution that consisted of 5% Tween 20 (Sigma-Aldrich, St. Louis, MO) dissolved in saline (0.9%) (*v/v*). All mice were injected intraperitoneally (i.p.) at a volume of 10 ml/kg, with drug solution, 30 min prior to behavioral testing.

### Forced swim test (FST)

For a more detailed description of this test and the OFT, see Fitzgerald et al.^32^. Briefly, two mice were typically tested simultaneously in a pair of clear Plexiglas cylinders, 30 cm high and 20 cm in diameter, filled halfway with water (24 ± 1 °C). An opaque white plastic divider was placed between the two forced swim tanks to block the animals’ view of one another. Mice were brought to the testing room in their homecages, and allowed to acclimatize to the room for ~1 h prior to testing. White lighting (300 lux) was present in the room during acclimation and throughout testing. At the start of each trial, the mouse was gently placed in the center of the tank and allowed to swim about freely. Each trial lasted 6 min, but behavior was only scored in the last 4 min^33^. During the test, movement of the animal was tracked with a camera system (mounted horizontally, facing the sides of the two tanks) and software package (EthoVision XT, Noldus Information Technology, Leesburg, VA). At the end of the trial, the mouse was immediately removed from the tank, dried off with a paper towel, and returned to its homecage. During later analysis, we defined “immobile” behavior in EthoVision as comprising frame-by-frame changes of 0–12% of pixels. “Mobile” (i.e., swimming) was defined as changes in 12–18% of pixels. “Highly mobile” (i.e., climbing) was defined as greater than 18% of pixels changing^32^.

### Open field test (OFT)

This test was carried out twice in “Experiment 5” (see below). Two mice were typically tested simultaneously, in neighboring open field boxes that had opaque white Plexiglas walls. Each box was cubic with 40 cm long walls and an open top. For analysis, the center region of the box floor was defined offline as a 20 × 20 cm square but was not marked. Each box was placed on the floor of the room, and was illuminated by indirect white lighting from tree lamps to ~40 lux in the corners and 80 lux in the center. At the start of each trial, the mouse was gently placed in a corner of the box, facing the center, and allowed to walk about freely. Each trial lasted 10 min and throughout this period, video was recorded for position and movement analysis. The camera was mounted vertically, centered above the two boxes, and EthoVision XT software package was used for both acquisition and subsequent analysis (Noldus Information Technology, Leesburg, VA). At the end of the trial, the mouse was immediately removed from the box and returned to its homecage. The box was cleaned with 70% ethanol solution and allowed to dry between animals. EthoVision was used to quantify the metrics of total distance traveled, center entries, and center time.

### Analysis and statistics

We first analyzed data with conventional parametric statistics (GraphPad Prism, GraphPad Software, La Jolla, CA). All FST data are shown in Table S1. Mice showing outlier behavior > 2 standard deviations from the mean (of the group for each drug dose) for a given FST or OFT behavior were excluded from that analysis (see Table S2 for number of animals removed from each experiment). One-way analysis of variance (ANOVA) followed by Dunnett’s multiple comparisons test were used to, respectively, determine if donepezil modulated FST or OFT behavior, and if so whether the drug groups differed significantly from the vehicle group in each test. Results are shown as mean ± standard error of the mean (SEM). Variance was similar between the groups that are being statistically compared.

We also carried out a randomization analysis to further investigate whether donepezil produced a u-shaped, or inverted u-shaped, dose-response curve for climbing, swimming, and immobile behavior in the FST. This analysis was carried out separately for each of these three behaviors in each FST session. For each analysis, we first calculated the observed mean difference between the average of the VEH and 2.0 mg/kg donepezil (DPZ 2.0) “outer” groups, versus the average of the 0.02 mg/kg and 0.2 mg donepezil “inner” groups (DPZ 0.02 and DPZ 0.2, respectively). Since there were eight mice in each of these four drug groups, we then shuffled these 32 values 100,000 times, while calculating for each shuffle the same mean difference between the average of the “outer” groups and the average of the “inner” groups, as noted above. A *p* value was then generated based on these 100,000 shuffles, by calculating the proportion of shuffles whose absolute value was as great or greater than the absolute value of the observed mean difference. In other words, and to be conservative in generating these *p* values, we tested for climbing, swimming, and immobility, whether there was either a “peak” or a “valley” for the two middle doses, relative to VEH or DPZ 2.0, that was larger than expected by chance. This is like using a two-tailed test rather than a one-tailed test, which made our *p* values twice as large.

## Results

In each of the five experiments (Expts 1–5) that comprised this study, we used a separate cohort of 32 mice to carry out a series of FST tests to investigate antidepressant-related properties of donepezil, as well as the OFT in Expt 5. A summary of the objectives and testing parameters of the five experiments is shown in Fig. S1, and the FST data from each mouse in all five experiments are shown in Table S1. We carried out multiple FSTs in each cohort of mice to determine if antidepressant-like effects would emerge after the first swim session. Each FST was separated by a week or more to allow almost all of the drug to be eliminated physiologically between tests. Orally administered donepezil has a plasma half-life of ~4 h in rats^34^, so after 1 week (i.e., about 42 half-lives) the plasma donepezil concentration should be <0.1% of its originally injected concentration. The 32 mice per cohort were divided into four groups of eight mice, one with each of our four administered doses. Importantly, rather than giving the same dose to each cohort of eight repeatedly, we often crossed over which cohort of eight mice got which dose of medication in each repeated dosing as shown in Fig. S2. This approach allowed us to uncover effects that were hidden with the first administration of donepezil.

### Experiment 1—repeated FSTs without crossing over drug cohorts

**Fig. 1:**
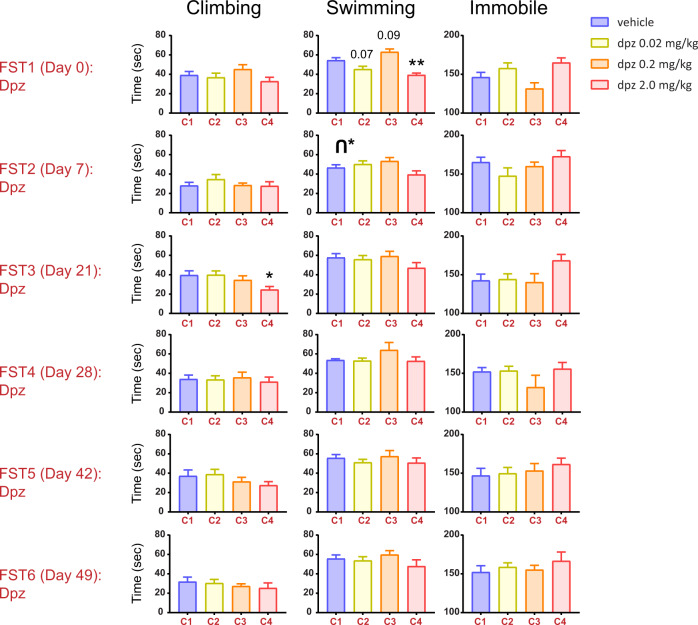
Repeated forced swims with the same donepezil groups do not show strong antidepressant-like effects. Shown are the six FSTs from Experiment 1, where a gap of 7 or 14 days was used for shorter versus longer washout periods, respectively, between swims. In this figure and the subsequent ones, statistically significant inverted u (climbing or swimming) or u-shaped (immobility) dose-response curves are depicted, based on their *p* values from the randomization test. Colored text (C1, C2, C3, and C4) below each bar denotes which cohort of eight mice was given that dose of donepezil for a given FST. Error bars represent mean ± SEM. For bars: *adjusted *p* < 0.05 versus vehicle, **adjusted *p* < 0.01 versus vehicle (or adjusted *p* value is explicitly shown for a statistical trend). *p < 0.05 for inverted u-shape.

In Expt 1, we gave C57BL/6J mice intraperitoneal donepezil at doses of 0.02 mg/kg, 0.2 mg/kg, the more traditional 2.0 mg/kg, or vehicle, followed by an FST test 30 min after injection (Fig. 1). (These three doses of donepezil, plus a vehicle group, were used in all experiments (Expts 1–5) in this study.) In this experiment, and unlike Fig. S2, we simply repeated the dosing of this medication in each cohort over multiple FSTs separated over weeks (FST1–6). The cohort IDs are indicated under each bar with C1, C2, C3, and C4 and are the same across all FSTs in this figure.

In FST1 of this first experiment, we found that donepezil did not statistically affect immobility behavior. It also had no significant effect on climbing behavior. On the other hand it modulated swimming (one-way ANOVA: F(3, 27) = 13.59; *p* < 0.01), with trending or significant depression-like effects at the low and high doses, with a contrasting antidepressant-like trend at the middle dose. In FST2, 7 days later, swimming behavior showed a trend toward modulation by donepezil dose (one-way ANOVA: F(3, 28) = 2.91; *p* = 0.052), driven by a mild increase in swimming (relative to vehicle) for the low and medium doses, but a decrease at the high dose (and an inverted u-shaped dose-response curve with our shuffling test; see Table S2). Immobility was not strongly modulated (*p* > 0.05), but qualitatively showed a u-shaped dose-response curve. We ran a specific shuffle test assessing for either u-shaped or inverted u-shaped dose-response curves and found that swimming behavior in FST2 demonstrated a more inverted-u-like profile than expected by chance (indicated by [inverted-u]* above the bars in that plot).

FSTs 3–6 tended to not show strong modulation by donepezil. Overall in Expt 1 repeated administration of the same doses to the same animals over multiple FSTs did not show robust modulation of FST behavior.

### Experiment 2—swapping dose-cohort pairings across trials

**Fig. 2:**
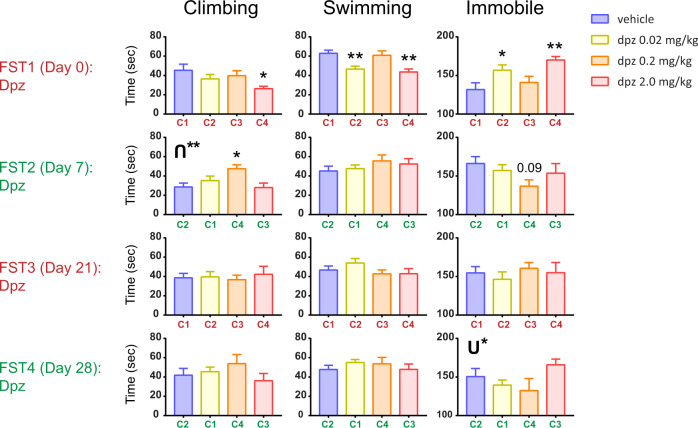
Crossing over drug groups in repeated forced swims yields antidepressant-like effects. Shown are the four FSTs from Experiment 2, where a gap of 7 or 14 days was used for shorter versus longer washout periods, respectively, between swims. After FST1, the vehicle and 0.02 mg/kg dose groups were switched (crossed over), as were the 0.2 and 2.0 mg/kg groups, for FST2. Such crossing over was also carried out in the subsequent FSTs, where maroon color indicates the original drug grouping, and green indicates the crossed over grouping. As shown, the maroon grouping (FST1 = FST3) was not significantly antidepressant-like to donepezil, whereas the green grouping (FST2 = FST4) was during FST2 (and also in FST4 based on the u-shaped randomization test). Error bars represent mean ± SEM. For bars: *adjusted *p* < 0.05 versus vehicle, **adjusted *p* < 0.01 versus vehicle (or adjusted *p* value is explicitly shown for a statistical trend). *p < 0.05 for u-shape, **p < 0.01 for inverted u-shape.

In the second experiment (Fig. 2), we sought to further study the effects seen in the literature, especially the possible antidepressant-like effects reported in some studies. We hypothesized that in Expt 1, we were not seeing large antidepressant-like effects due to the repeated dosing procedure we used. To investigate this in Expt 2, FST1 was the same as in Expt 1, but in subsequent swims we “crossed over” the drug groups as shown in Fig. S2: (1) animals previously given vehicle injections were given 0.02 mg/kg donepezil, and vice versa; (2) animals given 0.2 mg/kg were swapped with 2.0 mg/kg for FST2. We hypothesized that giving each mouse a dose that differed from what it received in FST1 would produce stronger antidepressant-like effects in subsequent swims.

FST1 immobility (Fig. 2) did not show an antidepressant-like effect of donepezil (but did show depression-like effects at the low and high doses) and qualitatively resembled FST1 from Expt 1. This finding was replicated in the swimming and climbing metrics.

In FST2, we crossed over the drug groups relative to FST1 (see markings of C1–C4 on the *x*-axis of Fig. 2 plots which signify animal cohorts; also see color-coded FST label texts at left of figure), and we observed qualitatively different results from FST1 of this experiment or all of Expt 1. First, without being significant in the one-way ANOVA, immobility showed a trend for an antidepressant-like reduction at the medium dose relative to vehicle (adjusted *p* = 0.086). Second, there was modulation of climbing behavior by drug (one-way ANOVA: F(3, 27) = 4.45; *p* < 0.05), where the medium dose showed an antidepressant-like effect relative to vehicle (adjusted *p* < 0.05).

Having found antidepressant-like effects or trends in FST2, we crossed the drug groups back over in FST3 to the FST1 groupings to see if antidepressant-like effects would emerge (note cohort numbers C1, C2, C3, and C4 are the same as in FST1 and there is the same color-coded FST label text). Like FST1, which had the same drug-cohort groupings, FST3 showed no significant antidepressant-like effects for climbing, swimming, or immobility (each *p* > 0.05).

In FST4, we then crossed back to match FST2, curious whether results similar to FST2 might emerge—and interestingly FST4 results did look qualitatively similar to FST2. These two tests shared the same cohort-dosage groupings and also similar trends in FST results across dosages, with the lower doses of donepezil showing actually reduced immobility relative to vehicle.

In fact, in FST4, this specific finding of 0.02 and especially 0.2 mg/kg being below vehicle and 2.0 mg/kg was verified by our statistical shuffling test for u-shaped dose-behavior effects: (U*) for immobility, with an inverted u-shape for climbing in FST2. This phenomenon is further tested in Expt 3.

### Experiment 3—Experiment 2 replication test

**Fig. 3:**
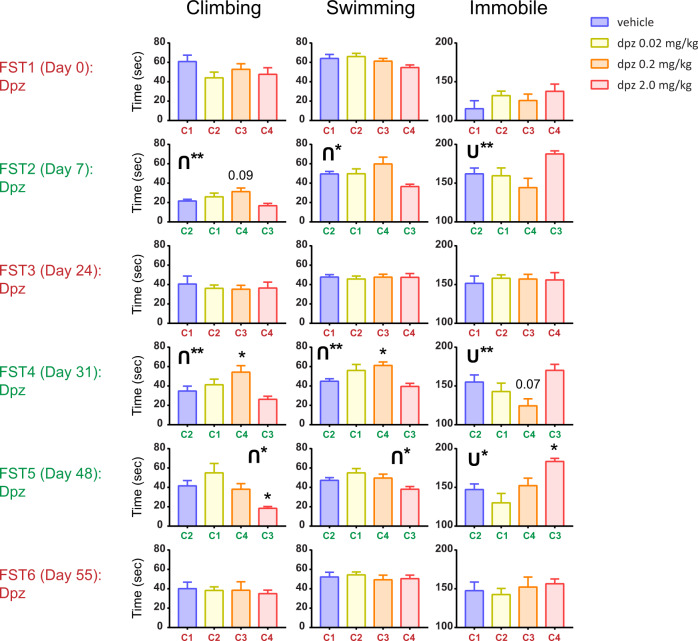
Extending the effects from Experiment 2 in a new cohort yields robust antidepressant-like effects. Shown are the results from Experiment 3. A gap of 7 or 17 days (slightly different from Experiment 2) was used for shorter versus longer washout periods, respectively, between FSTs. Maroon (FST1 = FST3 = FST6) indicates the original drug grouping, and green (FST2 = FST4 = FST5) indicates the crossed over grouping. Error bars represent mean ± SEM. For bars: *adjusted *p* < 0.05 versus vehicle (or adjusted *p* value is explicitly shown for a statistical trend). *p < 0.05 for u-shape or inverted u-shape, **p < 0.01 for u-shape or inverted u-shape.

In Expt 3 (Fig. 3), we sought to replicate the results seen in Expt 2, but we used an even longer delay between pairs of 7-day separated tests (17 days) to see if this might further amplify the antidepressant-like effects of the lower doses of donepezil.

In FST1, we again did not have strong antidepressant-like effects, with immobility not significantly modulated by drug (*p* > 0.05).

For FST2, we crossed over the drug groups as in Expt 2 (see color-coded FST label text on the left, and C1–4 on *x*-axis in Fig. 3). Immobility was modulated by drug (one-way ANOVA: F(3, 27) = 3.68; *p* < 0.05), as was climbing behavior (F(3, 24) = 4.14; *p* < 0.05), and comparison of each of the three drug groups with vehicle showed a trend toward the medium dose being elevated (adjusted *p* = 0.087). Drug treatment also affected swimming behavior (one-way ANOVA: F(3, 27) = 4.11; *p* < 0.05), but differences between vehicle and each drug group were not statistically significant. Importantly, we observed a u-shaped curve here as in Expt 2, FST4, signifying that the two lower doses showed lower immobility than the vehicle and high dose of donepezil (U** in Fig. 3).

For FST3, we crossed the drug groups back over to the FST1 groupings and again found no significant drug modulation for climbing, swimming, or immobility (each *p* > 0.05). Thus the same dosing per cohort as FST1 yielded a similar behavioral profile across cohorts as FST1.

For FST4, we then switched the doses per cohort back to the arrangement from FST2. The results were qualitatively similar to those from FST2, and revealed antidepressant-like effects at the middle and lower doses. One-way ANOVA showed that immobility, swimming, and climbing behaviors were all modulated by drug dose (immobility: F(3, 28) = 4.42; *p* < 0.05; climbing: F(3, 28) = 4.86; *p* < 0.01; swimming: F(3, 26) = 5.40; *p* < 0.01). The immobility in FST4 here again showed more u-like behavior than expected by chance.

Given the alternation of non-effects in FST1 and FST3 with antidepressant-like effects in FST2 and FST4, we next tested whether: (a) alternating trial sequencing or (b) cohort-drug matching was most crucial in these alternating findings. To do this we broke the alternating pattern by using the same groupings as FST4 for FST5. Note that the spacing between FSTs 2 and 3 was 17 days, while the other spacing were 7 days, so we wanted to investigate whether the antidepressant-like effects observed in FST2 and FST4 were “primed” by drug effects carrying over from the prior FST only a week before. Despite the fact that FST5 was carried out after a 17-day break and did not have an intervening test, the results showed antidepressant-like effects as in FST4. This indicates that the cohort by drug combination may be dictating FST results. Specifically, we found that in a one-way ANOVA, immobility was modulated by drug (F(3, 28) = 6.51; *p* < 0.01), with a significant depression-like increase in the high dose compared to vehicle (adjusted *p* < 0.05). Climbing and swimming behaviors also showed a main effect of drug with ANOVA (climbing: F(3, 28) = 5.71; *p* < 0.01), with the high dose showing less climbing than vehicle (*p* < 0.05); swimming: F(3, 27) = 3.86; *p* < 0.05. Once again, a u-shaped curve was found here to a degree greater than chance for immobility, with low doses of donepezil showing greater antidepressant-like effects than vehicle or high dose donepezil (U*, Fig. 3).

FST6 used the FST1 and FST3 drug groupings, and consistent with those groupings once again did not show modulation by drug. Therefore, FST5 and FST6 imply that the combinations of drug dose with cohort in later trials, rather than alternation, determined responses in the FST.

### Experiment 4—removing donepezil from FST1 to test necessity for observed effects in FST2

**Fig. 4:**
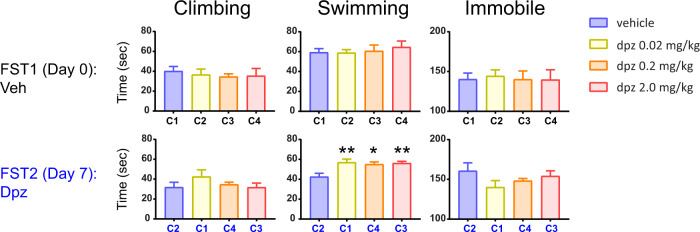
Prior exposure to donepezil is not necessary for its antidepressant-like effects observed in later swims. Shown are the results from Experiment 4, consisting of a new cohort of mice that was given vehicle only injections for FST1, followed 7 days later by donepezil treatment (FST2). Immobility shown here was qualitatively u-shaped, and swimming behavior showed antidepressant-like effects in FST2. Error bars represent mean ± SEM. For bars: *adjusted *p* < 0.05 versus vehicle, **adjusted *p* < 0.01 versus vehicle.

In the fourth experiment (Fig. 4), we continued to try to dissect the alternating nature of the findings in Expts 2 and 3. Here, we sought to address whether prior donepezil exposure in the FST is necessary for the antidepressant-like effects we observe in subsequent swims—or whether FST exposure in the absence of drug is sufficient. All mice received vehicle only injections during FST1, and their climbing, swimming, and immobile behavior did not differ across treatment groups (each one-way ANOVA: *p* > 0.05).

For FST2, mice received donepezil treatment for the first time. Climbing and immobile behavior were not strongly modulated by drug (each *p* > 0.05), but swimming was (one-way ANOVA: F(3, 25) = 5.81; *p* < 0.01), where the low and high doses (adjusted *p* < 0.01), as well as the medium dose (adjusted *p* < 0.05), were all significantly greater than vehicle. Thus, prior exposure to donepezil is not necessary for mice to exhibit antidepressant-like effects in the FST in our line of experiments.

### Experiment 5—open field testing to determine generalized locomotion with donepezil

**Fig. 5:**
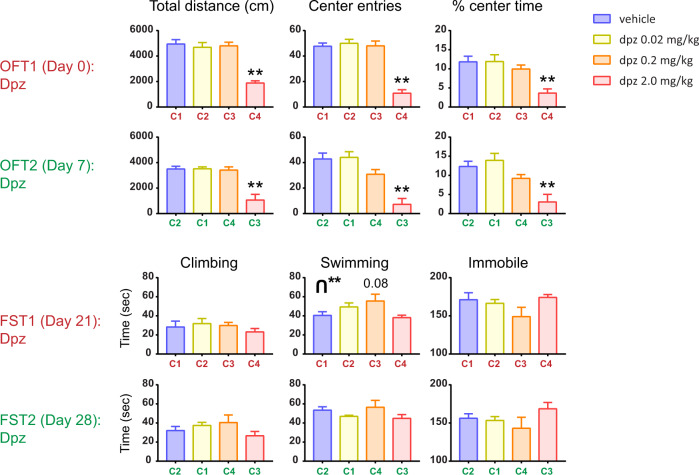
Antidepressant-like effects of low dose donepezil are not due to generalized hyperactivity. Shown are the results from Experiment 5, consisting of a new cohort of mice that was given the open field test (OFT) twice, followed by two FSTs. A gap of 7 or 14 days was used for shorter versus longer washout periods, respectively, between OFTs or FSTs. Crossing over of drug groups was the same as in Experiments 2 and 3 (maroon grouping: OFT1 = FST1; green grouping: OFT2 = FST2). Error bars represent mean ± SEM. For bars: **adjusted *p* < 0.01 versus vehicle (or adjusted *p* value is explicitly shown for a statistical trend). **p < 0.01 for inverted u-shape.

In the fifth experiment (Fig. 5), we sought to address whether the antidepressant-related effects we observed in the FST of the first four experiments are confounded by generalized changes in locomotion on drug, and also whether donepezil modulates anxiety-related behavior. This is of particular interest given the association between locomotive or attentional states and acetylcholine^35^. So we used an OFT as a basic test of locomotion and tendency to move in mice, which also measures anxiety-related behavior. We also replicated our alternating dose versus cohort design to study the effects of that manipulation in the OFT.

In the first OFT for these mice (OFT1), only the highest dose of donepezil showed a large effect, generally reducing mobility (one-way ANOVA: F(3, 27) = 20.88; *p* < 0.01). OFT1 center square entries showed similar results (one-way ANOVA: F(3, 27) = 36.77; *p* < 0.01), with the high dose markedly lower than the other groups (adjusted *p* < 0.01). Percent center square time, which is more a measure of anxiety-related behavior than locomotion, also showed drug modulation (one-way ANOVA: F(3, 28) = 8.03; *p* < 0.01), with the high dose showing what may be an anxiogenic-like effect (adjusted *p* < 0.01). However, given the large decrease in total distance traveled for the highest dose, it is difficult to determine if percent center square time really does reveal an anxiogenic-like effect.

We then crossed over the drug groups (to mimic the previous FST experimental designs) and ran OFT2. The results were qualitatively similar to OFT1, with no large effects of drug except at the high dose, and none increasing locomotion.

We also investigated, in this same cohort of animals, whether they would exhibit similar FST behavior to Expts 1–4. To test this, we crossed the drug groups back to match OFT1 and ran FST1 14 days after OFT2. In FST1, immobile (while qualitatively being mildly u-shaped) and climbing behavior were not significantly modulated by donepezil but swimming showed a strong trend (one-way ANOVA: F(3, 28) = 2.92; *p* = 0.052), driven by the medium dose trending toward an antidepressant-like effect relative to vehicle (adjusted *p* = 0.081). It should be noted that the results here in FST1 qualitatively resemble FST2 of Expts 2 and 3, suggesting that prior experience with being injected and being run in either the FST or OFT may facilitate the emergence of antidepressant-like behavior in subsequent FSTs. We next crossed over the drug groups and ran FST2, which did not yield any significant modulation by drug. Thus, FST1 here qualitatively showed a u-like shape for immobility similar to Expts 1–4, whereas the OFT in this same cohort did not show this, seemingly pointing to capacities of these two tests to discriminate specific behavioral elements.

## Discussion

Here we have shown that acute administration of low doses of the acetylcholinesterase inhibitor, donepezil, has antidepressant-like effects in the C57BL/6J mouse FST in some circumstances. These effects are not limited to decreases in immobility, as we also found increases in climbing or swimming in various instances. These therapeutic effects are not confounded by generalized hyperactivity as shown by the OFT, and also are not accompanied by anxiolytic-like effects. Furthermore, these effects tend to emerge after two or more FST sessions, and their expression may relate to specific learned associations or history of dosages (more below). In those sessions where we observed an antidepressant-like response, we typically find a u-shaped dose-response curve wherein a high donepezil dose reduces overall mobility (pro-depressant like) and lower doses have an opposite antidepressant-like effect.

Initially it appeared that our treatment effect may be alternating from trial to trial. These trials had different temporal spacing and so we tried to disrupt the alternations (FST5-6 of Expt 3) and found that it was not the sequence but rather the cohort/drug dose history that predicted the response to new doses of donepezil. We suggest here that the mice may have formed a learned association between the initially highly aversive testing conditions of FST1 and the interoceptive state induced by that dose of donepezil. In this scenario, when the mice were again presented with the same doses of donepezil in FST3 and FST6 of Expt 3 (and FST3 of Expt 2), they tended to not express an antidepressant-like effect. However, as the perceived aversiveness of the injections and FST itself may have decreased with subsequent exposures (FST2 and beyond), and the mice were presented with a new dose of drug in FST2, they then exhibited an antidepressant-like effect that could be replicated with the same doses in FST4–5.

While the more standard dose of 2.0 mg/kg did not tend to show antidepressant-like effects, both the medium dose (0.2 mg/kg) and low dose (0.02 mg/kg) of donepezil showed antidepressant-like effects in various experiments. The medium dose was typically therapeutic in earlier swims than the low dose. Expt 4 further demonstrates that it is probably not “priming” effects of previous exposure to donepezil that dictate its antidepressant-like effects in a subsequent FST session, since we found antidepressant-like effects in FST2 of this experiment when during FST1 only vehicle injections were administered. Rather the exposure to the first FST itself or to OFT in Expt 5 seemed to have an “unlocking” effect on certain cohorts in the subsequent FST.

### Relation to prior work

As noted in the “Introduction”, we are not the first lab to report antidepressant-like effects of cholinesterase inhibitors in rodents. Papp et al. have demonstrated that rats exposed to chronic mild stress and treated chronically with the cholinesterase inhibitors rivastigmine or donepezil showed an antidepressant-like response to both drugs in the sucrose preference test^17^. It is interesting to note that the dose of donepezil (0.3 mg/kg) used in that study is similar to our medium dose (0.2 mg/kg), which frequently had antidepressant-like effects in our experiments. Another study reported acute antidepressant-like properties of donepezil, in their case in the mouse FST^18^. Unlike in our study, they used Swiss mice and also reported an antidepressant-like response at much higher doses of this drug (up to 30 mg/kg). Further they found the cholinesterase inhibitors rivastigmine and tacrine lacked this response and went on to suggest that the antidepressant-like response of donepezil was not mediated through cholinesterase inhibition but rather involved the sigma-1 receptor. Their study also did not find a depression-like response to donepezil at their higher doses, unlike in our study. Our interpretation of their study is that there may be large mouse strain differences, including C57BL/6J versus Swiss mice, in the therapeutic effects of donepezil. In addition, the very high doses used in those experiments may have meant that different receptor systems were the basis of their effects, such as the sigma-1 system and therefore their relevance to this study is not clear. Chronic treatment with rivastigmine in olfactory bulbectomized mice, a rodent model of depression, has also been shown to be antidepressant-like in various tests including the FST^19^.

The findings we report here may be largely consistent with the cholinergic-adrenergic hypothesis of mood disorders^36^, but require a modification of it. That theory posits that a low ratio of cholinergic to adrenergic signaling promotes mania, whereas a high ratio promotes depression. While not all of our data showed a depression-like effect at the highest dose of donepezil we used (2 mg/kg), we did in most cases observe an increase in immobility at this dose relative to our low and medium doses. These findings may support the cholinergic-adrenergic hypothesis at a high dose of donepezil. On the other hand, at lower doses (and possibly lower concentrations of synaptic acetylcholine) cholinergic signaling can have antidepressant-like effects only under certain conditions, such as repeated testing. We suggest that since the antidepressant-like effects of donepezil may not be strongly present in the first swim exposure and may even be depression-like in that swim (Figs. 1–3), other researchers have typically reported depression-like effects of cholinesterase inhibitors such as physostigmine in the FST or tail suspension test^10–13,37,38^. The “longitudinal” forced swim procedure we carried out here, with multiple swim exposures, was implemented out of necessity and allowed for replication of the effects of previous swims (and antidepressant-like effects) within the same cohort of animals. Since male C57BL/6J mice are known to exhibit habituation (i.e., increased immobility) upon repeated exposure to the FST^32,39,40^, the antidepressant-like effects that we observed here with donepezil in subsequent swims suggest that this drug still increases active behaviors in this test relative to vehicle even though there may be a tendency for all groups to habituate in the absence of drug. We found that the largest antidepressant-like effects may not have been present in the second swim either, and can emerge in later swims (Fig. 3). Our data may also be consistent with cholinergic-adrenergic functional opposition in another way: the presumably high perceived stressfulness of the first injection and swim exposure may include elevated stress hormone (adrenergic and noradrenergic) signaling that counteracts the ability of donepezil to induce an antidepressant-like effect. Regarding the lack of robust drug efficacy during the first swim exposure; it should be noted that the rat FST typically uses an initial pretest swim exposure to accentuate drug effects during a second swim^41^. It has also been demonstrated in rats that repeated FSTs, spaced 7 or 14 days after the initial two swims, may reliably detect short- and long-term effects of SSRIs or other antidepressant drugs^42^.

The data presented here are consistent with the view that synaptic acetylcholine has an inverted u-shaped relationship with mood, where low to moderate boosting of the tonic concentration of this neurotransmitter with donepezil has antidepressant-like properties, whereas a high degree of boosting is depression-like. (See Table S2 for additional analyses on this topic.) Grasing has suggested a similar inverted u-shaped model for acetylcholine in substance abuse^43^, and the dose-response curve for physostigmine may also be inverted u-shaped in rats performing a water maze task^44^. If such a relationship exists for acetylcholine in mood regulation and substance abuse, it would extend the ideas of Arnsten, Giustino, Maren, and colleagues that various neurotransmitters, such as norepinephrine and dopamine, have inverted u-shaped functional characteristics, where an “optimal” amount of signaling is most healthy, and too much or too little transmission may be pathological for cognitive- and emotion-related behavior^45–47^.

It should also be noted that the C57BL/6J mouse strain that we used here exhibits atypical anxiety-related behavior in assays such as the light-dark exploration test after chronic restraint stress^48,49^. These atypical anxiety-related responses of this strain, relative to other inbred strains, also extend to the elevated plus maze^48^ and OFT, where it was shown to be unresponsive to benzodiazepine anxiolytics^50^. The C57BL/6J strain has also been characterized as relatively stress resilient^51–53^, and is known to show either therapeutic resistance or atypical responses to the behavioral effects of SSRIs^49,54^. Given these response characteristics of C57BL/6J mice to various pharmacological agents in acutely stressful tests such as the FST and OFT, it is plausible that other inbred mouse strains would deviate from the depression-related and anxiety-like findings we report here for donepezil.

Cholinesterase inhibitors, such as donepezil, galantamine, and rivastigmine, are currently widely used to treat the severe and prevalent neurological disorder, Alzheimer’s disease, which is characterized by loss of cholinergic neurons in the basal forebrain^55,56^. It may seem surprising that donepezil (and possibly other cholinesterase inhibitors) may also have antidepressant properties, given both the apparent unrelatedness of MDD and Alzheimer’s and how frequently this drug is used. But individuals with Alzheimer’s often have comorbid MDD, and the SSRI citalopram ameliorates cognitive impairment in Alzheimer’s to some extent^57^, suggesting that there may be pathophysiological overlap between the two diseases. Cholinesterase inhibitors have been reported to exhibit antidepressant properties in elderly patients with Alzheimer’s disease^58,59^, although several studies of older adults with MDD did not find therapeutic effects of these drugs, suggesting they may have limited efficacy in geriatric depression at least^16,60,61^. In addition, we specifically report here that the antidepressant properties emerge from lower doses of this drug, that are likely far below those of what most patients receive.

Donepezil and other cholinesterase inhibitors have also been shown to exhibit therapeutic effects on the behavioral and pathophysiological consequences of traumatic brain injury (TBI). These favorable effects on TBI have been reported both in human subjects and in rodent models^62–65^. While not all of the studies have found therapeutic effects, either in rodents or humans^66,67^, a body of literature supports their efficacy under some conditions^68^, including demonstration in a double-blind placebo-controlled study of donepezil^69^.

In summary, here we have shown that the cholinesterase inhibitor, donepezil, has antidepressant-like properties in the C57BL/6J mouse FST. These findings may have translational ramifications, in that donepezil could be repurposed as an antidepressant in human subjects. This topic should now be investigated further, including developing a greater understanding of the modulation of this drug’s therapeutic effects through associative learning processes, with an eye toward testing in human subjects suffering from MDD.

## Supplementary Information

Fig. S1

Fig. S2

Table S1

Table S2

Supplementary Information
