# Supplementary material for: The cholinesterase inhibitor donepezil has antidepressant-like properties in the mouse forced swim test
Source: Transl Psychiatry. 2020 Jul 25;10:255. doi: 10.1038/s41398-020-00928-w (PMC7382650; doi:10.1038/s41398-020-00928-w)
Supplement: Supplementary file 1 — Fig. S1 [file 41398_2020_928_MOESM1_ESM.pdf]

	Principal question	Drug groups	Number of tests	Gap between tests
Experiment 1	Does donepezil (Dpz) have antidepressant-like effects in the repeated FST?	Kept constant	6 FSTs	7 or 14 days
Experiment 2	Does crossing over the drug groups amplify antidepressant-like effects in the repeated FST?	Crossed over	4 FSTs	7 or 14 days
Experiment 3	Can we replicate Expt 2 but with a slightly longer gap (17 days) to possibly amplify effects?	Crossed over	6 FSTs	7 or 17 days
Experiment 4	Is prior administration of Dpz necessary for antidepressant-like effects in subsequent swims?	Not applicable	2 FSTs	7 days
Experiment 5	Are antidepressant-like effects of Dpz confounded by generalized hyperactivity in the OFT?	Crossed over	2 OFTs, 2 FSTs	7 or 14 days
