# Supplementary figures and images for: The cholinesterase inhibitor donepezil has antidepressant-like properties in the mouse forced swim test
Source: Transl Psychiatry. 2020 Jul 25;10:255. doi: 10.1038/s41398-020-00928-w (PMC7382650; doi:10.1038/s41398-020-00928-w)

Immobile

- vehicle
- dpz 0.02 mg/kg
- dpz 0.2 mg/kg
- dpz 2.0 mg/kg

FST1 (Day 0):  
Dpz

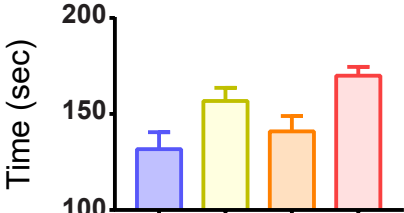

FST2 (Day 7):  
Dpz

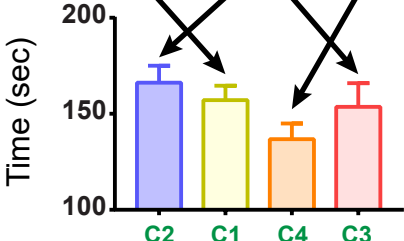

FST3 (Day 21):  
Dpz

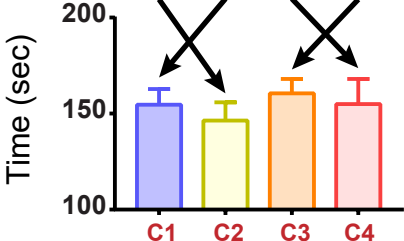

FST4 (Day 28):  
Dpz

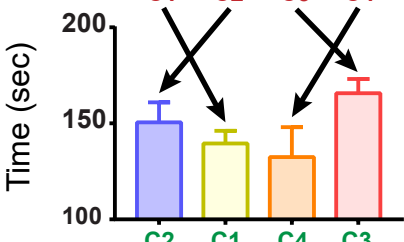

Supplement: Supplementary file 2 — Fig. S2 [file 41398_2020_928_MOESM2_ESM.pdf]
