# Supplementary material for: The cholinesterase inhibitor donepezil has antidepressant-like properties in the mouse forced swim test
Source: Transl Psychiatry. 2020 Jul 25;10:255. doi: 10.1038/s41398-020-00928-w (PMC7382650; doi:10.1038/s41398-020-00928-w)
Supplement: Supplementary file 5 — Supplementary Information [file 41398_2020_928_MOESM5_ESM.docx]

*Supplementary Information*

Randomization Analysis

As described in the Materials and Methods, we also carried out a randomization analysis to further investigate whether donepezil produced a u-shaped, or inverted u-shaped, dose-response curve for climbing, swimming, and immobile behavior in the FST. Table S2 shows the p values generated from this analysis, carried out with the same > 2 standard deviations outliers removed as in Figures 1-5, while also showing the results with no outliers removed. In general, the results support an inverted u-shaped relationship between the four drug groups for climbing and swimming behavior in FSTs where the drug was effective, such as FST2, FST4, and FST5 of Expt 3. Likewise, this analysis tends to support a u-shaped curve for immobility in the same responsive FSTs. Unresponsive FSTs, on the other hand, tend not to show these relationships, such as FST1, FST3, and FST6 of Expt 3.

*Supplemental Legends*

Table S1. Raw forced swim data from all five experiments. These data were automatically calculated over the last four minutes of the six minute FST, with our camera and software package, EthoVision XT. Data are parsed into the three mutually exclusive behaviors: climbing, swimming, and immobility time (seconds).

Table S2. Randomization analysis reveals u-shaped or inverted u-shaped dose-response curves on donepezil for climbing, swimming, and immobile behavior in the FST. See Materials and Methods for details on the approach used. Shown are all the p values generated in this analysis, with or without > 2 standard deviation outlier values removed. Also shown are the number of animals excluded from each FST session and behavior, based on that criterion.

Figure S1. Summary of objectives and parameters used in Experiments 1-5.

Figure S2. Illustration of the drug cohort crossover technique used in Experiments 2, 3 and 5. The example shown here is from Expt 2, where during FST1, cohort 1 (C1: n=8 mice) received a vehicle injection, cohort 2 (C2) received 0.02 mg/kg donepezil, cohort 3 (C3) received 0.2 mg/kg, and cohort 4 (C4) received 2.0 mg/kg. As shown with the arrows in this figure, the drug doses given to C1 and C2 were swapped for FST2, as were C3 and C4. This means that during FST2, C1 received 0.02 mg/kg, C2 got vehicle, whereas C4 got 0.2 mg/kg and C3 got 2.0 mg/kg. For FST3, the drug groupings were crossed back to the original FST1 groupings. For FST4, the groupings were crossed again to match the FST2 groupings. Note for FST1 and 3, the maroon coloring of C1-4 on the x axis, and the labels to the left of each graph, indicating the same drug groupings in these two FSTs. FST2 and 4 are instead colored green to show that their groupings are the same.
